# Molecular Integrative Study on Inhibitory Effects of Pentapeptides on Polymerization and Cell Toxicity of Amyloid-β Peptide (1–42)

**DOI:** 10.3390/cimb46090606

**Published:** 2024-09-14

**Authors:** Lianmeng Ye, Nuela Manka’a Che Ajuyo, Zhongyun Wu, Nan Yuan, Zhengpan Xiao, Wenyu Gu, Jiazheng Zhao, Yechun Pei, Yi Min, Dayong Wang

**Affiliations:** 1 Key Laboratory of Tropical Biological Resources of the Ministry of Education of China, School of Pharmaceutical Sciences, Hainan University, Haikou 570228, China; 20071000110006@hainanu.edu.cn (L.Y.);; 2 Laboratory of Biopharmaceuticals and Molecular Pharmacology, One Health Cooperative Innovation Center, Hainan University, Haikou 570228, China; 3 Department of Biotechnology, School of Life and Health Sciences, Hainan University, Haikou 570228, China

**Keywords:** Alzheimer’s disease, Aβ_42_, polymerization, inhibitor, pentapeptides, molecular docking, molecular dynamics, pharmacology

## Abstract

Alzheimer’s Disease (AD) is a multifaceted neurodegenerative disease predominantly defined by the extracellular accumulation of amyloid-β (Aβ) peptide. In light of this, in the past decade, several clinical approaches have been used aiming at developing peptides for therapeutic use in AD. The use of cationic arginine-rich peptides (CARPs) in targeting protein aggregations has been on the rise. Also, the process of peptide development employing computational approaches has attracted a lot of attention recently. Using a structure database containing pentapeptides made from 20 L-α amino acids, we employed molecular docking to sort pentapeptides that can bind to Aβ_42_, then performed molecular dynamics (MD) analyses, including analysis of the binding stability, interaction energy, and binding free energy to screen ligands. Transmission electron microscopy (TEM), circular dichroism (CD), thioflavin T (ThT) fluorescence detection of Aβ_42_ polymerization, MTT (3-[4,5-dimethylthiazol-2-yl]-2,5 diphenyl tetrazolium bromide) assay, and the flow cytometry of reactive oxygen species (ROS) were carried out to evaluate the influence of pentapeptides on the aggregation and cell toxicity of Aβ_42._ Two pentapeptides (TRRRR and ARRGR) were found to have strong effects on inhibiting the aggregation of Aβ_42_ and reducing the toxicity of Aβ_42_ secreted by SH-SY5Y cells, including cell death, reactive oxygen species (ROS) production, and apoptosis.

## 1. Introduction

Alzheimer’s disease (AD) is a progressive neurodegenerative disease associated with damaged neurons in the brain that is consistent with an irreversible neurodegenerative disorder involving a decline in memory and executive function, and personality change (Hansen, et al. [1]). On a global scale, it is escalating at a daunting rate. There are approximately 6.7 million individuals with AD dementia in the United States and an estimated 50 million individuals with AD dementia globally. Due to an increase in the aging population and since Alzheimer’s disease is prevalent amongst the elderly, these populations are expected to grow to 12.7 million and 150 million in the United States and globally by 2050 [2,3].

Amyloid-beta (Aβ) is a 39–43 amino acid residue peptide and a major constituent of extracellular amyloid plaque, and its expression is believed to be a major contingency in AD advancement [4]. Aβ is the peptide outcome of the consecutive proteolytic cleavages (by β- and ϒ-secretases) of APP (a type-I transmembrane protein). These proteolytic cleavages give rise to two types of Aβ isoforms (Aβ_40_ and Aβ_42_), and though Aβ_40_ is more profuse than Aβ_42_ in human fluids, Aβ_42_ aggregates faster and is considered to be more neurotoxic than Aβ_40_. Aβ is distributed throughout the prefrontal cortex, hippocampus, midbrain, lower brainstem, and cerebellar cortex. It was reported that Aβ oligomers may bind to receptors on neuronal membranes, inducing calcium influx, which leads to calcium overload and cytotoxic responses [5]. Moreover, they may can interact with synaptic proteins, disrupting synaptic transmission and plasticity, ultimately causing synaptic dysfunction [6]. The oxidative stress toxicity of Aβ, alterations in mitochondrial function, and metal ion toxicity have also been widely reported [7,8]. Oxidative stress causes damage to lipids, proteins, and DNA, including antioxidant enzymes. Aβ aggregates may induce excessive production of ROS produced by mitochondria or disruption of metal ion homeostasis.

Peptide-based inhibitors, among numerous inhibitors, have been corroborated to be possible anti-aggregation agents due to their low cytotoxicity, good biocompatibility, high target affinity, and specificity [9,10,11,12,13,14,15,16].

Computational techniques have been used to uncover prospective inhibitors against numerous biological targets during the past few years [17,18]. Diverse contemporary studies have delineated and assessed prospective peptides as inhibitors of numerous targets using computational methods [19,20,21,22,23,24].

Two vital goals in drug discovery are developing molecules that bind tightly to a target protein and weakly—or not at all—to off-target proteins. In recent years, a group of rigorous, physics-based methods have surfaced as the most consistently precise method that can aid in the discovery of promising compounds and help hasten the slow and expensive process of lead development [25,26,27,28]. One of these methods is free energy (Gibbs free energy).

Gibbs free energy is equal to the sum of the enthalpy plus the product of the temperature and entropy of the system under the conditions of constant pressure and temperature. The change in Gibbs free energy (∆G) determines whether a reaction will happen spontaneously. Calculating the binding free energy of a compound to a protein target has emerged to be beneficial for drug discovery. Using the molecular dynamics (MD) method established [29], we conducted an initial analysis of the thermodynamic mechanisms of Aβ_42_ polymerization [30]. It was found that Aβ_42_ monomer is a globular protein; however, Aβ_42_ strands stably maintain an S-shape polymerized structure while the number of Aβ_42_ strands is more than five. Hydrophobic contact is the predominant force piloting Aβ_42_ clustering by lowering free energy, and three hydrophobic regions in the structure affect polymerization in that replacing the hydrophobic side chains of the amino acids in these regions with that of glycine differentially decreased the binding free energy [30]. Since the three hydrophobic regions determine polymerization, chemicals that can bind to the regions will interfere with polymerization. Based on this notion, we performed molecular docking and MD analysis to screen pentapeptides targeting the hydrophobic regions using a structure database consisting of all pentapeptides (3,200,000) made from the 20 L-α-amino acids, and conducted pharmacological experiments to test the effects of the pentapeptides.

## 2. Materials and Methods

### 2.1. Materials

Pentapeptides were synthesized by Sangon Biotech (Shanghai, China), and dissolved in 10 mM phosphate buffer (pH 7.4). Aβ_42_ was bought from ChinaPeptides (QYAOBIO) (Shanghai, China) and DMSO used to dissolve it to a concentration of 1 mM. All allotting solutions were aliquoted and preserved at −80 °C until use. DMSO, ThT, and Lipofectamine 2000 were bought from Sigma-Aldrich (St. Louis, MI, USA). PEI 40K transfection reagents were bought from Servicebio (Wuhan, China). An ROS active oxygen testing kit and apoptosis kit for the detection by flow cytometry were bought from Bioscience (Shanghai, China).

### 2.2. Molecular Docking

Python was used to summon the ChemScript module of the ChemDraw software version 20.0 (Shelton, CT, USA), and then a database of pentapeptides made from 20 L-α amino acids was constructed. The molecular structural data file of Aβ_42_ (PDB# 5OQV) was downloaded from the Protein Data Bank (PDB). After correcting the data and adding missing hydrogen atoms, the open-source software AUTODOCK was used to investigate the interaction of pentapeptides with Aβ_42_. The London algorithm for free energy was used for screening the binding sites and conformations of pentapeptides and then refined by the generalized-Born volume integral/weighted surface area algorithm.

### 2.3. Molecular Dynamics Analysis of Binding Stability

Molecular dynamic analysis (MDA) has been extensively used in drug design [31] for the physical and chemical depiction of intricate movements between biomolecules [32,33,34]. MDA enables us to have a better understanding of the dynamic attributes of biomolecular systems, such as protein folding and stability, ligand binding, and protein complexing, among others. A key feature of MDA is its ability to imitate both in vitro and in vivo conditions—for example, at different pH conditions, in the presence of water and ions, at different salt or ionic concentrations, and in the presence of a lipid bilayer and other cellular components [35,36]. MDA has been used to study multiple protein-related issues, such as protein-binding and protein–protein interaction and signaling [35,37].

In our research, molecular dynamics studies were carried out with the Groningen Machine for Chemical Simulation (GROMACS, 2020.03) on the Ubuntu (18.06) Linux operating system, and were sped up by NVIDIA Compute Unified Device Architecture (CUDA)-supported parallel computation. The Aβ_42_ monomer, trimer, or pentamer was centralized in a dodecahedron box with a distance of 3.0 nm from the edge to the Aβ_42_ molecule. The box was filled with water molecules, and Na^+^ and Cl^−^ counter ions at final concentrations of 0.1 M were added to the box to keep the system overall neutral at physiological pH. Throughout the MD study, we employed the Amber99SB force field (which is optimized for the ab initio calculation of three-dimensional structure of proteins) and the TIP3P explicit water model. Energy minimization and system equilibration was carried out with the same formulae as previously reported by [30].

### 2.4. Umbrella Sampling of Binding Free Energy

The binding free energy is mirrored in alterations in Gibbs free energy (∆G) in an isothermal–isobaric assemblage throughout the procedure of hauling oligopeptides off Aβ_42_. The Aβ_42_ protein complex fastened to the pentapeptide was placed at dimensions 3.0 × 3.5 × 1.5 (x, y, z) nm in a cubic box of dimensions 6.0 × 7.0 × 14.0 (x, y, z) nm. The periodic boundary conditions were employed in all simulations. Water molecules were added into the cubic box, and 0.1 M sodium chloride was also added along with the number of Na^+^ and C^−^ counter ions essential to maintain an overall neutral system at physiological pH. The carbonyl carbon atom (Cα) of the 29th glycine and the Cα of every pentapeptides’ second amino acid were chosen as reference atoms. Pressure equilibration was implemented before the pulling and umbrella sampling phases. During the phase of generating configurations, the two proteins were pulled away when a harmonic force at a constant velocity of 0.01 nm/ps over a course of 250,000-time steps was utilized, and 501 coordinate files were saved in the course of the pulling procedure. A total of 23 to 25 umbrella samplings of 10 ns were conducted in each overlapping 0.2 nm spacing sampling window through the reaction axis (ξ), bringing about approximately 450 Gb of data. The GROMACS’s WHAM module was used to determine ∆G.

### 2.5. ThT Fluorescent Detection of Aggregation of Aβ_42_

Amyloid aggregation was evaluated using the amyloid dye Thioflavin-T (ThT) [38]. Aβ_42_ (QYAOBIO, Shanghai, China) was dissolved as previously reported by Yuan et al. [30]. The Aβ_42_ was adjusted to a final concentration of 10 μM, the pentapeptides (TRRRR and ARRGR) adjusted to a final concentration of 40 μM, and ThT adjusted to a final concentration of 50 μM. These were all carried out in a 96-well plate. The fluorescence intensity was measured at 37 °C using an automated well plate reader (TECAN Infinite 200 PRO, Tecan Asia Pte Ltd., Singapore) at an emission wavelength of 485 at a 5 min interval, with an excitation wavelength of 450 nm. The measurements were performed as independent quintuplicates.

### 2.6. Transmission Electron Microscope Observation of Aggregated Aβ_42_

Aβ_42_ (QYAOBIO, Shanghai, China) samples were prepared in PBS at a working concentration of 50 μM. Aβ_42_ was incubated with TRRRR and ARRGR 37 °C for 48 h. The final concentration of Aβ_42_ was 10 μM, and that of TRRRR and ARRGR was 40 μM. After incubation, 10 μL of samples to be observed were dropped onto a 300-mesh Formvar–carbon-coated copper grid and stained with 10 μL of 2% phosphotungstic acid solution. Afterwards, the samples were air-dried and observed under a TEM (FEI Inc., Hillsboro, OR, USA) with a voltage of 200 kV.

### 2.7. Cell Culture

SH-SY5Y cells were expanded in DMEM including 10% fetal bovine serum supplemented with glutamine, and grown at 37 °C with 5% CO_2_. When the cells reached 80% confluency, they were used for further experiments.

### 2.8. Transfection and Expression of Secretable Aβ_42_

Before transfection, the medium was replaced with Opti-MEM (Gibco, Billings, MT, USA) and incubated for 2 h. Then, a mixture of pcDNA3.1-Aβ_42_ plasmids (4.0 μg, containing the sequence encoding the signal peptide for secretion at the 5′ end of Aβ_42_ sequence and three consecutive stop codons at the 3′ end; Aβ_42_ sequence: 5′-ATGCTGCCCGGTTTGGCACTGCTCCTGCTGGCCGCCTGGACGGCTCGGGCGGATGCAGAATTCCGACATGACTCAGGATATGAAGTTCATCATCAAAAATTGGTGTTCTTTGCAGAAGATGTGGGTTCAAACAAAGGTGCAATCATTGGACTCATGGTGGGCGGTGTTGTCATAGCGTGATGATGA-3′). Lipofectamine 2000 (10 μL, Sigma, St. Louis, MI, USA) and Opti-MEM that had been pre-incubated for 15 min at room temperature was added into the plates and incubated for 4 h at 37 °C. Four hours after the transfection, the Opti-MEM was replaced with DMEM (ThermoFisher, Waltham, MA, USA) containing 5% FBS and antibiotics (penicillin and streptomycin). After transfection, G418 was supplemented 24 h later at a final concentration of 100 µg/mL for positive selection.

### 2.9. ELISA Experiment

TRRRR or ARRGR was added to the cells secreting Aβ_42_ at final concentrations of 10 µM or 50 µM 4 h after transfection. A total of 24 h later, 100 μL of the supernatant was transferred to 96-well high-absorbent plates and incubated at 4 °C overnight. The wells were then washed four times with PBS, followed by blocking with 5% non-fat milk for 2 h. Primary antibody (1:200) was added and incubated at 4 °C overnight. After four washes with PBS, HRP-conjugated secondary antibody (1:1000) was added. Following treatment with the EL-TME kit (C520026, Sango Biotech, Shanghai, China), chemiluminescence was measured by using a microplate reader.

### 2.10. Microscopic Observation of Cell Death

TRRRR or ARRGR was added to the cells secreting Aβ_42_ 4 h after transfection at final concentrations of 10 µM or 50 µM, followed by incubation at 37 °C for 24 h. The culture medium was removed, and the cells were fixed with 4% paraformaldehyde at room temperature for 15 min. The cells were gently washed twice with PBS, followed by incubation with EB (1 mg/mL) at 37 °C in the dark for 20 min. The cells were gently washed twice with PBS, then observed under a fluorescence microscope (Axio Observer 7, Zeiss, Germany) (excitation wavelength: 545 nm, and emission wavelength: 590 nm).

### 2.11. Flow Cytometry Methods to Detect Reactive Oxygen Species and Apoptosis

After the Opti-MEM was replaced with DMEM medium containing 5% FBS and antibiotics (penicillin and streptomycin), SH-SY5Y cells expressing Aβ_42_ were incubated with TRRRR or ARRGR at final concentration of 10 μM and 50 μM for 24 h. After collecting and washing cells with PBS, they were stained using PI and the Annexin-V kit (UElandy Inc., Suzhou, China), then cell apoptosis was computed by flow cytometry (CytoFLEX LX, Beckman Coulter Life Sciences, Indianapolis, IN, USA).

### 2.12. Statistical Analysis

The results of experiments were expressed as mean ± SD when applicable. Differences among groups have been evaluated by One-way ANOVA, followed by the Tukey-Kramer test for multiple comparisons using GraphPad Prism 6.0 (Boston, MA, USA). A *p* value less than 0.05 was considered statistically significant.

## 3. Results

### 3.1. Molecular Docking of Pentapeptides

**Figure 1:**
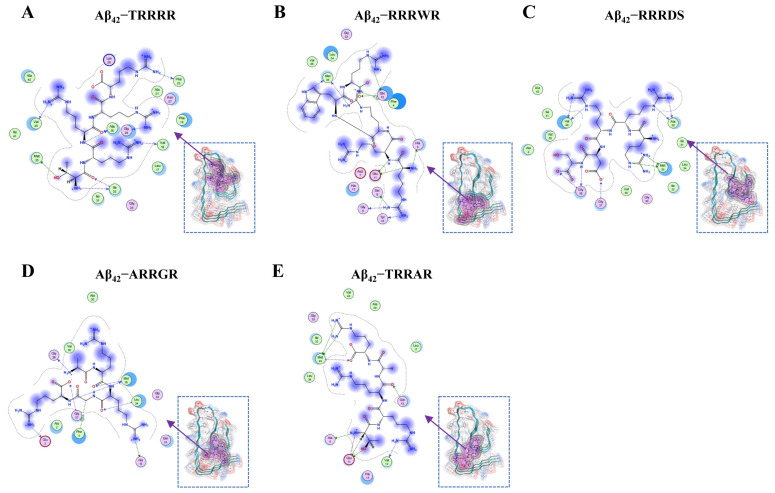
Molecular docking of the five pentapeptides with Aβ_42_. (**A**) Binding of TRRRR with Aβ_42_. (**B**) Binding of RRRWR with Aβ_42_. (**C**) Binding of RRRDS with Aβ_42_. (**D**) Binding of ARRGR with Aβ_42_. (**E**) Binding of TRRAR with Aβ_42_. For clarity, only three strands taken from Aβ_42_ pentamer are shown in this figure. The magenta mesh represents the molecular surface of the pentapeptides, and the gray mesh represents the boundary of van der Waal’s force. Arrows indicate the hydrogen bonds. TRRRR: Threonine-arginine-arginine-arginine-arginine; RRRWR: Arginine-arginine-arginine-tryptophan-arginine; RRRDS: Arginine-arginine-arginine-aspartic acid-serine; ARRGR: Alanine-arginine-arginine-glycine-arginine; TRRAR: Threonine-arginine-arginine-alanine-arginine.

Hydrophobic interaction and electrostatic forces including hydrogen bonds have an immense influence on the conformational alterations of Aβ_42_ [39]. In this study, a molecular docking experiment was conducted between Aβ_42_ and five pentapeptides. The pentapeptides which were incorporated in the Aβ_42_ hydrophobic cluster (Figure 1) were Threonine-arginine-arginine-arginine-arginine (TRRRR), Arginine-arginine-arginine-tryptophan-arginine (RRRWR), Arginine-arginine-arginine-aspartic acid-serine (RRRDS), Alanine-arginine-arginine-glycine-arginine (ARRGR), and Threonine-arginine-arginine-alanine-arginine (TRRAR). The docking free energy of the five peptides are shown in Supplementary Table S1. As illustrated on Figure 1, the binding of pentapeptides to Aβ_42_ can be seen. The grids in magenta denote the molecular surfaces of the pentapeptides, whereas those in gray denote the range of van der Waals forces.

But for the RRRWR pentapeptide, which did not interact with any of the hydrophobic patches of the Aβ_42_ monomer, the other four pentapeptides had side-chain interactions with the Aβ_42_ monomer via hydrophobic clusters through hydrogen bonds including π-type bonding. On the incorporation of RRRWR in the Aβ_42_ monomer, few side-chain contacts were noted at the N-terminal (6–13) region. However, contacts between CHC (17–22)/mid-domain (26–32) and C-terminal (34–42) regions were reduced. As for the incorporation of RRRDS in the Aβ_42_ monomer, few side-chain contacts were noted at the hydrophobic patch on the C-terminal (30–35) region. For the incorporation of TRRAR in the Aβ_42_ monomer, few side-chain contacts were noted at the N-terminal (6–15) and central hydrophobic core (Leucine 17) regions. Also, few side-chain contacts were noted at the N-terminal (13, 15) and the hydrophobic patch at the C-terminal (33–35) regions when ARRGR was incorporated in the Aβ_42_ molecule. Finally, for TRRRR and Aβ_42_, few side-chain contacts were noted at the central hydrophobic core of Aβ_42_ (17–21) and the hydrophobic patch towards the C-terminal (30–33) regions. However, based on the other experiments conducted, TRRRR and ARRGR had better inhibitory effects on the polymerization of Aβ_42_ when compared to the other three peptides. So, the reports of the remaining experiments focused on these two peptides (TRRRR and ARRGR).

**Figure 2:**
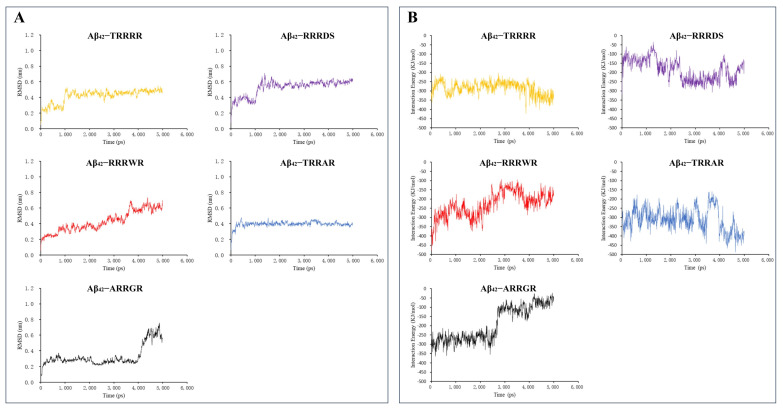
The binding stability and interaction energy of the pentapeptides at different hydrophobic regions. (**A**) The binding stability of pentapeptides to Aβ_42_ pentamer. (**B**) The interaction energy between Aβ_42_ pentamer and the pentapeptides. RMSD: The root mean square deviation of the positions of the heavy elements of a pentapeptide. The interaction energy is the algebraic sum of Lennard-Jones and Coulombic potential energy. TRRRR: Threonine-arginine-arginine-arginine-arginine; RRRWR: Arginine-arginine-arginine-tryptophan-arginine; RRRDS: Arginine-arginine-arginine-aspartic acid-serine; ARRGR: Alanine-arginine-arginine-glycine-arginine; TRRAR: Threonine-arginine-arginine-alanine-arginine.

**Figure 3:**
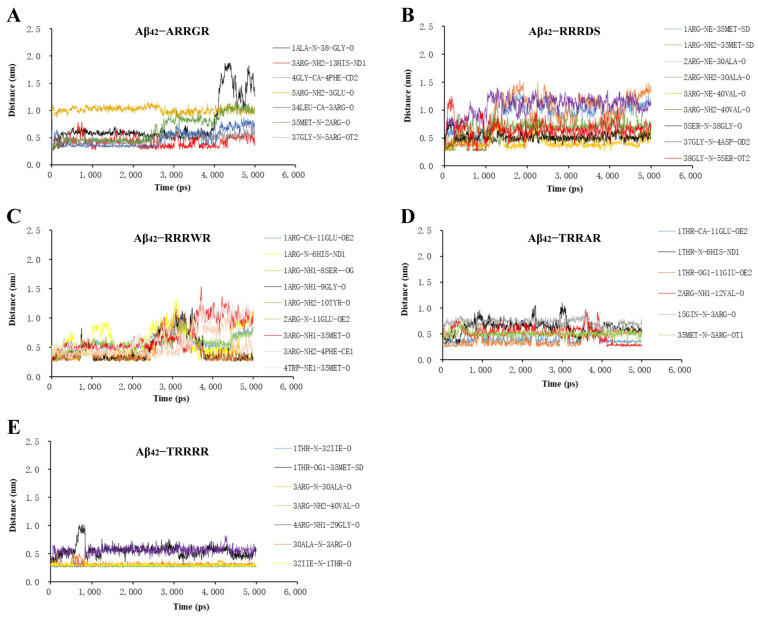
Variation in the lengths of hydrogen bonds formed between the pentapeptides and Aβ_42_. (**A**) ARRGR and Aβ_42_. (**B**) RRRDS and Aβ_42_. (**C**) RRRWR and Aβ_42_. (**D**) TRRAR and Aβ_42_. (**E**) TRRRR and Aβ_42_. TRRRR: Threonine-arginine-arginine-arginine-arginine; RRRWR: Arginine-arginine-arginine-tryptophan-arginine; RRRDS: Arginine-arginine-arginine-aspartic acid-serine; ARRGR: Alanine-arginine-arginine-glycine-arginine; TRRAR: Threonine-arginine-arginine-alanine-arginine.

**Figure 4:**
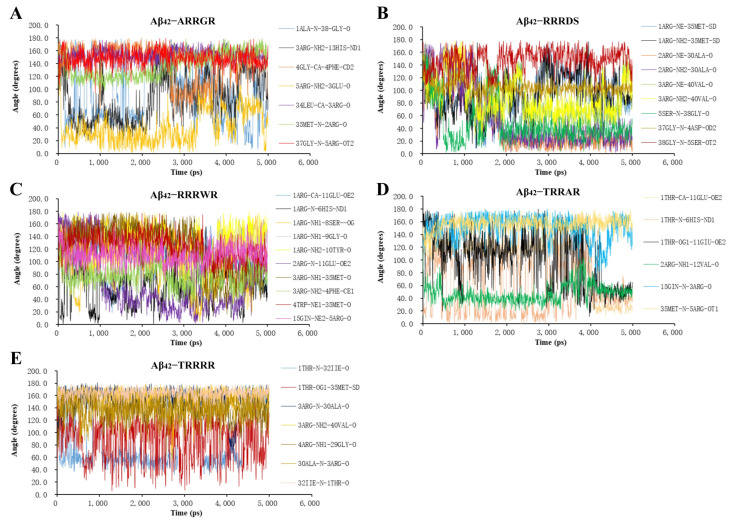
Variation in the angles of hydrogen bonds formed the pentapeptides and Aβ_42_. (**A**) **A**RRGR and Aβ_42_. (**B**) RRRDS and Aβ_42_. (**C**) RRRWR and Aβ_42_. (**D**) TRRAR and Aβ_42_. (**E**) TRRRR and Aβ_42_. TRRRR: Threonine-arginine-arginine-arginine-arginine; RRRWR: Arginine-arginine-arginine-tryptophan-arginine; RRRDS: Arginine-arginine-arginine-aspartic acid-serine; ARRGR: Alanine-arginine-arginine-glycine-arginine; TRRAR: Threonine-arginine-arginine-alanine-arginine.

The RMSD fluctuated at a higher value after 1.0 ns and then steadily attained a stable plateau (Figure 2A). The average RMSD of Aβ_42_ pentamer displayed limited change in the presence of pentapeptides which indicates stable binding of the pentapeptides with the Aβ_42_ pentamer. Also, the interaction energy of pentapeptides with Aβ_42_ (Figure 2B) was relatively stable. The conformational change of Aβ_42_ is mainly affected by electrostatic and hydrophobic interaction forces including hydrogen bonds [40]. The hydrogen bond formed when Aβ_42_ interacts with ligand can effectively prevent the generation of hydrogen bond between Aβ_42_^−^Aβ_42_ and inhibit the formation of Aβ_42_ aggregates [41]. Hydrogen bonds can form hydrogen bonds over long distances in the range of 0.5 nm. The gmx distance calculates the distance between two locations as a function of time. Each selection specifies an independent set of distances to be calculated. The gmx distance instruction was executed in the protein–ligand interaction analysis window of GROMACS (2020.03) to run the distance between atoms of hydrogen bond interaction between tripeptide and pentapeptides ligands and Aβ_42_ monomer. The results of the hydrogen bond distances are shown in Figure 3 and Figure 4. The distances of the five pentapeptides were all less than 2.0 nm in 5 ns time. The hydrogen bond distance between TRRRR and the atoms of the Aβ_42_ docking complex (Figure 3E) was maintained at about 0.3 nm and 0.6 nm, and the interacting hydrogen bond was generally stable. The hydrogen bond distance formed between the atoms of ARRGR and Aβ_42_ bonding complex (Figure 3A) changes by microwave during the running time of 4 ns to 5 ns, showing a tendency to increase the hydrogen bond distance, suggesting that the binding between ARRGR and Aβ_42_ may be unstable; further molecular biology experiments are needed to verify this.

The gmx angle can be used to calculate the angle distribution of multiple angles or dihedral angles. A hydrogen bond is easier to form when the angle between the donor atom, hydrogen atom, and acceptor atom is greater than 120° [42]. The index file command can be executed to specify hydrogen bond atoms and the gmx angle command can be run to calculate the angle between the atoms of hydrogen bond interacting between a pentapeptide ligand and Aβ_42_. The results of the angles of the hydrogen bonds are shown in Figure 4. The hydrogen bond angles formed between the atoms of ARRGR or TRRRR and the Aβ_42_ docking complex (Figure 4A,E) are mostly greater than 120°, which means that hydrogen bond formation between pentapeptide ARRGR or TRRRR and Aβ_42_ monomer is relatively easy. It may affect the formation of hydrogen bond between Aβ_42_^−^ Aβ_42_ to potentially inhibit Aβ_42_ aggregation.

**Figure 5:**
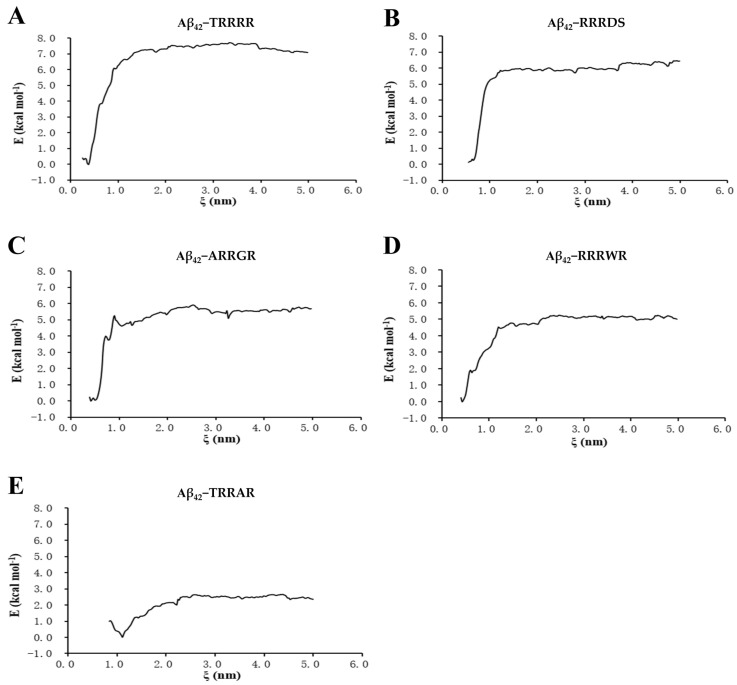
Binding free energy between the pentapeptides and Aβ_42_. (**A**) TRRRR and Aβ_42_. (**B**) RRRDS and Aβ_42_. (**C**) ARRGR and Aβ_42_. (**D**) RRRWR and Aβ_42_. (**E**) TRRAR and Aβ_42_. TRRRR: Threonine-arginine-arginine-arginine-arginine; RRRWR: Arginine-arginine-arginine-tryptophan-arginine; RRRDS: Arginine-arginine-arginine-aspartic acid-serine; ARRGR: Alanine-arginine-arginine-glycine-arginine; TRRAR: Threonine-arginine-arginine-alanine-arginine.

From results obtained from the umbrella sampling free analysis of peptides binding to Aβ_42,_ TRRRR had the highest free binding energy curve (Figure 5), indicating that it bound more firmly to Aβ_42_ compared to the other peptides. The higher energy value indicates that more energy was required to pull TRRRR from Aβ_42._ After TRRRR, RRRDS and ARRGR also had higher binding energies than RRRWR and TRRAR.

### 3.2. Effects of Pentapeptides on Aggregation of Aβ_42_ Detected by ThT Fluorescence Assay

**Figure 6:**
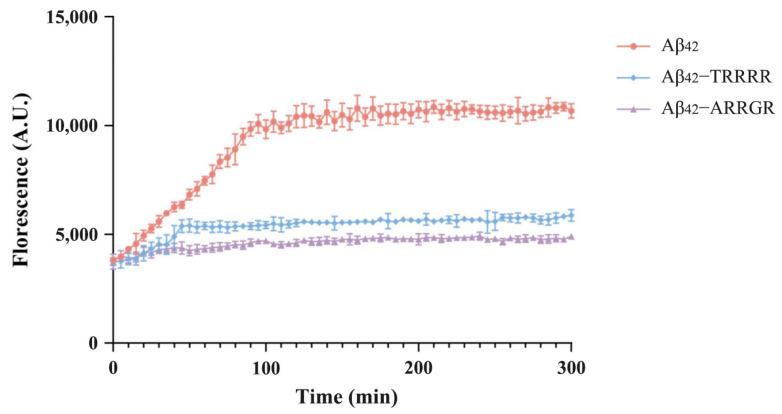
Effects of pentapeptides on aggregation of Aβ_42_ detected by ThT fluorescence assay. Results are expressed as means ± SD, *p* < 0.01 among groups, tested by two-way ANOVA, n = 5.

From the results obtained from our computational analysis of the interaction between pentapeptides and Aβ_42_, TRRRR and ARRGR were chosen for further experiments with the ThT fluorescence assay. The fluorescence intensity of TRRRR and ARRGR were evaluated independently to determine their inhibitory effects on Aβ_42_ aggregation. Also, there was an Aβ_42_ group for the control experiment. The fluorescence intensity of the Aβ_42_ control group was higher than that of TRRRR and ARRGR after it was incubated solo at 37 °C for 48 h (Figure 6). Those of the groups of TRRRR incubated with Aβ_42_ and ARRGR incubated with Aβ_42_ were lower. The high fluorescent intensity of the Aβ_42_ control group could be attributed to the fact that Aβ_42_ amassed a large quantity of ThT. The decline in fluorescence intensity in the groups containing TRRRR and ARRGR could be attributed to a decrease in the quantity of Aβ_42_ clusters. These definitely demonstrate that our cationic arginine-rich pentapeptides may be effective at inhibiting the polymerization of Aβ_42_.

### 3.3. Observation of the Effect of TRRRR or ARRGR on Aβ_42_ Aggregation with a Transmission Electron Microscope (TEM)

**Figure 7:**
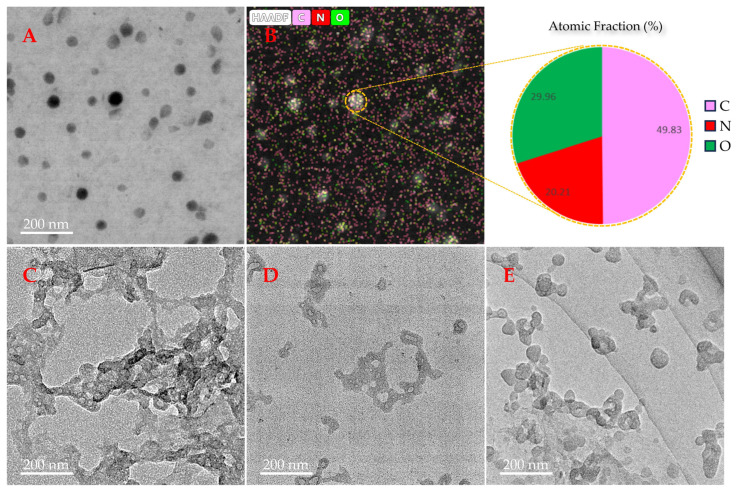
Effect of pentapeptides on aggregation of Aβ_42_ detected by transmission electron microscopy. (**A**) Transmission electron microscopic images of 10 μM Aβ_42_ before incubation. (**B**) The Atomic Fraction of Aβ_42_ detected by HAADF-STEM imaging. (**C**) Transmission electron microscopic images of 10 μM Aβ_42_ incubated for 48 h. (**D**) Transmission electron microscopic images of 10 μM Aβ_42_ co-incubated with 40 μM TRRRR for 48 h. (**E**) Transmission electron microscopic image of 10 μM Aβ_42_ co-incubated with 40 μM ARRGR for 48 h.

A transmission electron microscope (TEM) was used to observe the effects of TRRRR or ARRGR on the aggregation of Aβ_42_. Many fibrils were clearly visible after incubating Aβ_42_ alone for 48 h (Figure 7C). Aβ_42_ incubated for 48 h too with TRRRR and ARRGR revealed minute clusters which did not have a well-defined shape (Figure 7D,E). Results from the transmission electron microscope showed that creation of Aβ_42_ fibrils were inhibited by both pentapeptides. This is supported by the fact that the growth of Aβ_42_ fibrils was inhibited to an extent.

### 3.4. ELISA Experiments to Investigate the Effects of TRRRR and ARRGR on Aβ_42_ Expression

**Figure 8:**
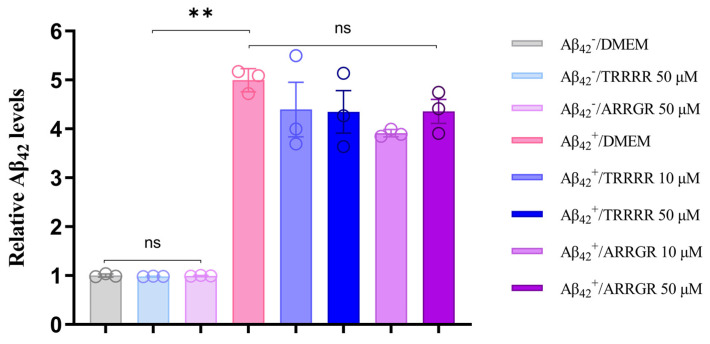
Effects of TRRRR and ARRGR on Aβ_42_ protein expression. Aβ_42_^–^: SH-SY5Y cells not secreting Aβ_42_; Aβ_42_^+^: SH-SY5Y cells secreting Aβ_42_. The results are expressed as means ± SD; ns: insignificant; ** *p* < 0.01; the results were analyzed by one-way ANOVA, followed by the Tukey–Kramer test for multiple comparisons, with n = 3.

To investigate the effects of TRRRR and ARRGR on Aβ_42_ protein expression, an ELISA experiment was conducted (as shown in Figure 8). The results indicated that Aβ_42_ protein endogenously expressed in SH-SY5Y cells was detected in the control group (SH-SY5Y cells not secreting Aβ_42_), TRRRR 50 μM treatment group, and ARRGR 50 μM treatment group, with no significant differences in expression levels observed among the groups. The Aβ_42_ levels in the Aβ_42_ group (SH-SY5Y cells secreting Aβ_42_) were significantly higher than in the aforementioned three groups. Additionally, no significant differences were observed among the Aβ_42_ group, Aβ_42_ with TRRRR 10 μM treatment group, Aβ_42_ with TRRRR 50 μM treatment group, Aβ_42_ with ARRGR 10 μM treatment group, and Aβ_42_ with ARRGR 50 μM treatment group. These findings suggest that pentapeptides do not affect Aβ_42_ protein expression level in SH-SY5Y cells.

### 3.5. Effect of Either TRRRR or ARRGR on Cell Death Induced by Secreted Aβ_42_

**Figure 9:**
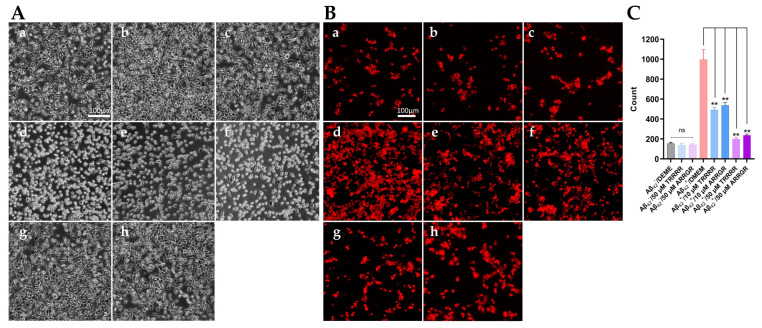
Effects of TRRRR and ARRGR against cell toxicity of Aβ_42_ secreted from SH-SY5Y. (**A**) The morphology of SH-SY5Y cells secreting Aβ_42_. Round-shaped cells with a bright edge are dying or dead. (**B**) Damaged cells detected with ethidium bromide, a nuclei acid tracer that cannot pass through an intact cell membrane. (**C**) Quantification of cell damage by using Image J. Results are expressed as means ± SD with ** *p* < 0.01, and were analyzed by one-way ANOVA, followed by the Tukey–Kramer test for multiple comparisons, n = 3; ns: insignificant. In (**A**,**B**): (**a**) SH-SY5Y control cells that do not secrete Aβ_42_. (**b**) The control cells treated with TRRRR at 50 μM. (**c**) The control cells treated with ARRGR at 50 μM. (**d**) SH-SY5Y cells secreting Aβ_42_. (**e**) Aβ_42_-secreting SH-SY5Y cells treated with 10 μM TRRRR. (**f**) Aβ_42_-secreting SH-SY5Y cells treated with 10 μM ARRGR. (**g**) Aβ_42_-secreting SH-SY5Y cells treated with 50 μM TRRRR. (**h**) Aβ_42_-secreting SH-SY5Y cells treated with 50 μM ARRGR.

Using a fluorescent microscope, the viability results of Aβ_42_-secreting SH-SY5Y cells in the control group and the pentapeptide treatment groups are presented in Figure 9. Ethidium bromide can incorporate into the DNA of dead cells that have lost their membrane integrity, resulting in the red-stained cells. The morphology of transfected SH-SY5Y cells treated with either TRRRR or ARRGR was not different from the negative control, indicating that they had no obvious cytotoxic effects on SH-SY5Y cells (Figure 9C). As shown in Figure 9B(d), a large number of SH-SY5Y cells that were transfected and secreted Aβ_42_ died. SH-SY5Y cells treated with 10 µM of the pentapeptides showed improved cell survival (Figure 9B(e,f)). With the treatment of 50 µM pentapeptides, the number of cell deaths was significantly reduced compared to the Aβ_42_-secreting group (Figure 9B(g,h)), indicating that either TRRRR or ARRGR could well ameliorate the cytotoxicity of Aβ_42_ secreted from SH-SY5Y cells.

### 3.6. Effect of Either TRRRR or ARRGR on Reactive Oxygen Species Produced by SH-SY5Y Cells Secreting Aβ_42_

**Figure 10:**
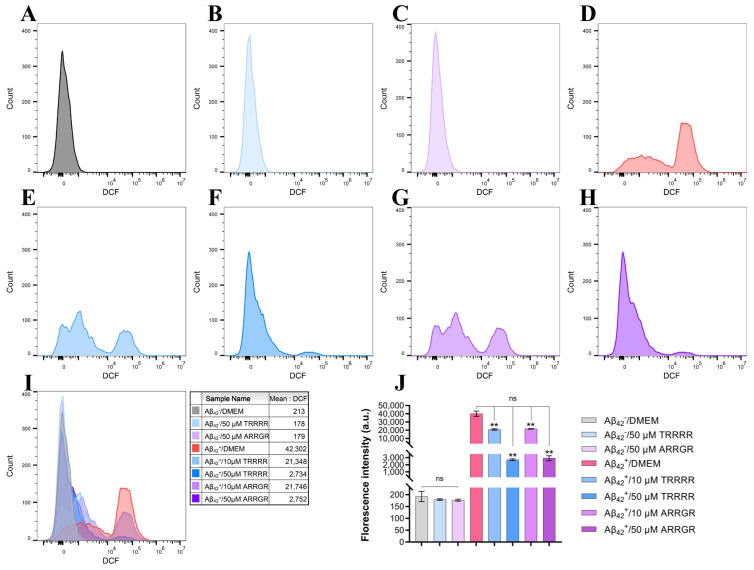
Effects of the pentapeptides on ROS levels in SH-SY5Y cells secreting Aβ_42_. (**A**) ROS levels in the SH-SY5Y control cells that do not secrete Aβ_42_. (**B**) ROS levels in the control cells treated with 50 μM TRRRR. (**C**) ROS levels in the control cells treated with 50 μM ARRGR. (**D**) ROS levels in SH-SY5Y cells secreting Aβ_42_. (**E**) ROS levels in the Aβ_42_-secreting cells treated with 10 μM TRRRR. (**F**) ROS levels in the Aβ_42_-secreting cells treated with 10 μM ARRGR. (**G**) ROS levels in the Aβ_42_-secreting cells treated with 50 μM TRRRR. (**H**) ROS levels in the Aβ_42_-secreting cells treated with 50 μM ARRGR. (**I**) Overlay of the flow cytometry plots (**A**–**H**). (**J**) Quantification of the ROS levels in the cells. Results are expressed as means ± SD, ns: not significant, ** *p* < 0.01, by one-way ANOVA, followed by the Tukey–Kramer test for multiple comparisons, with n = 3.

SH-SY5Y cells secreting Aβ_42_ and those not secreting Aβ_42_ were incubated independently with either TRRRR or ARRGR for 24 h. After this, a fluorescent probe DCFH DA on a flow cytometer was used to compute the intracellular ROS levels. According to the results obtained (Figure 10), ROS levels in SH-SY5Y cells expressing secreted Aβ_42_ were significantly reduced by TRRRR and ARRGR, respectively, compared to non-treated SH-SY5Y cells secreting Aβ_42_. Also, the influence of TRRRR and ARRGR were dose-dependent.

### 3.7. Protective Effect of Either TRRRR or ARRGR on Cell Apoptosis of SH-SY5Y Cells Secreting Aβ_42_

**Figure 11:**
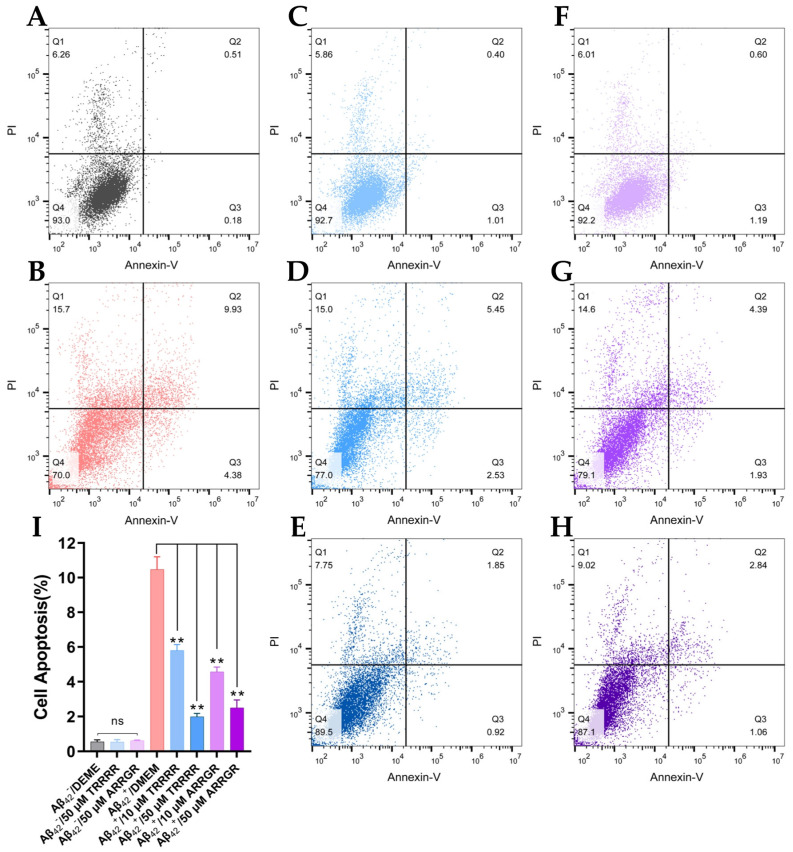
Effects of pentapeptide on apoptosis of SY-SY5Y cells secreting Aβ_42_. (**A**) Apoptosis in the SH-SY5Y control cells that do not secrete Aβ_42_. (**B**) Apoptosis in the control cells treated with 50 μM TRRRR. (**C**) Apoptosis in the control cells treated with 50 μM ARRGR. (**D**) Apoptosis in SH-SY5Y cells secreting Aβ_42_. (**E**) Apoptosis in the Aβ_42_-secreting cells treated with 10 μM TRRRR. (**F**) Apoptosis in the Aβ_42_-secreting cells treated with 10 μM ARRGR. (**G**) Apoptosis in the Aβ_42_-secreting cells treated with 50 μM TRRRR. (**H**) Apoptosis in the Aβ_42_-secreting cells treated with 50 μM ARRGR. (**I**) Overlay of the flow cytometry plots (**A**–**H**). (**J**) Quantification of apoptosis in the cells. Results are expressed as means ± SD; ns: not significant, ** *p* < 0.01; results were analyzed by one-way ANOVA, followed by the Tukey–Kramer test for multiple comparisons, with n = 3.

SH-SY5Y cells were categorized by different treatments with an apoptosis kit and then evaluated by flow cytometry, and the results can be seen in Figure 11. Figure 11I shows the quantification of cell apoptosis. In Figure 11, Q1 denotes nude nucleus necrotic cells, Q2 denotes necrotic and late apoptotic cells, Q3 early apoptotic cells, and Q4 living cells. In the group which received drug dosages (pentapeptides), cell apoptosis was significantly reduced when either TRRRR or ARRGR were added (Figure 11I). This was particularly significant when the drug dosage was at 50 μM as compared to the group which did not receive the pentapeptides. These results prove that both TRRRR and ARRGR can effectively protect cells from cytotoxicity.

## 4. Discussion

Amyloid plaques are generated when Aβ is produced excessively, not cleared after production, and then form clusters. These clusters also promote the inflammation of brain tissues and apoptosis. The first pathological occurrence that happens years before the development of clinical symptoms is probably going to be the accumulation of Aβ in the brain. Aβ is generated when there is proteolytic cleavage of the amyloid precursor protein (APP). APP is a transmembrane glycoprotein, which has a cytoplasmic β-domain with 55 amino acids and an extracellular domain with 590–680 amino acids [43]. When the proteases β- and ϒ-secretases cleave APP, different sizes of Aβ fragments are generated, according to the cleavage sites [44]. Aβ_40_ which is approximately 90% of the Aβ fragments generated, and Aβ_42_ which is approximately 5–10% of the Aβ fragments generated, are the most preeminent. However, Aβ_42_ is more toxic than Aβ_40_. After the generation of the Aβ fragments, they coalesce to form amyloid clusters. These clusters are in disparate configurations such as low-molecular-weight oligomers, protofibrils, and mature fibrils.

The principal therapeutic targets investigated for AD have been directly or indirectly concomitant to neurofibrillary clusters (tau protein) and Aβ senile plaques (protein) [45,46]. Most of these therapeutic targets are developed with the aim of inhibiting aggregation and fostering the clearance of fibrils. However, investigations on inhibitors such as the β-secretase 1 (BACE-1) and ϒ secretases have been discontinued due to challenges in toxicity [45,47,48,49]. Nevertheless, there have been efforts put into developing metal ion-chelating agents [50], peptides [51], natural compounds [52], and biomolecules [53,54]. Peptides have drawn a lot of scrutinization and the number of approved peptide biotherapeutics has been on the rise over the contemporary decades. This has been an interesting strategy because they have the ability to bind with larger interfacial pockets than small molecules [55]. Also, the agenda of peptide development has been piloted by significant approaches in computational structural prognosis and the augmentation of available chemical refinements to ameliorate affinity, stability, and specificity. Peptides are considered a better option for AD than small molecule-based compounds because of their high affinity for Aβ and low toxicity [56]. With this knowledge, peptides could be potential candidates for inhibiting conformational transitions, self-assembly, and toxicity against neurons, and the promotion of the pathways of the nontoxic fibrillation and early diagnosis of Alzheimer’s disease [57]. Our laboratory previously reported the inhibitory effects of dipeptides [30] and tripeptides [58] on Aβ polymerization. We made a discovery that, through hydrophobic interactions between β strands, the Aβ_42_ monomer first aggregates into oligopeptides and while they lengthen, clumps are formed through parallel clustering. The whole process of the formation of clusters is piloted by hydrophobic interactions and stabilized by electrostatic forces.

Here, in the present work, we demonstrated the inhibitory effects of the cationic arginine-rich peptides (CARPs) TRRRR and ARRGR on Aβ polymerization [10,59,60], performed a detailed review of peptide-based inhibitors developed for AD, and pointed out that, regardless of their advantages, the lack of membrane permeability and difficulty in penetrating the blood–brain barrier are obstacles to the development of anti-aggregatory peptides. Different schools of thought, however, have shown that cationic arginine-rich peptides (CARPs) can demonstrate a special ability to cross membranes of the cell and the blood–brain barrier [61,62,63], thus making them more commonly used ‘‘carrier’’ molecules for a variety of therapeutic ‘‘cargo’’ such as oligonucleotides, peptides, and proteins (reviewed by [64]).

In addition, it has been suggested that there are multiple potential mechanisms for CARPs to confer cytoprotecting against toxic aggregates of amyloid-beta. As described, the predominant approach has focused on inhibiting aggregation; CARPs which have demonstrated the ability to reduce amyloid-beta-induced cytotoxicity in vitro by inhibiting aggregation include those developed by [65,66,67,68].

Arginine has many attributes that make it truly special, to a great extent due to the peculiar structure and chemistry of the guanidinium group [69]. Not only have arginine monomers long been considered a formidable inhibitor of protein aggregation [70], polypeptides containing many arginine residues also influence the formation and cytotoxicity of protein clusters in a fascinating manner. Arginine residues have several significant bioactive properties that are very effective in regulating protein aggregation [71,72,73], and should therefore be taken into consideration when developing Aβ-targeting therapeutic peptides for treating Alzheimer’s disease. Pentapeptides containing arginine bound more tightly to Aβ_42_ monomer as compared to histidine-containing pentapeptides due to the electrostatic interactions of arginine with the negatively charged residues of the Aβ_42_ monomer [74].

Aβ peptide (1–42) fibrils are organized as β-sheets [75,76]. Aβ peptide aggregation pathways are determined by the primary amino acid sequence and intermolecular interactions [30,77]. Aβ monomers can form higher-order fabrications ranging from low-molecular-weight oligomers, including dimers, trimers, tetramers, and pentamers, to midrange-molecular-weight oligomers, including hexamers, nanomers, and dodecamers, to protofibrils and fibrils [77]. When molecular dynamics analysis was used to probe mechanisms underlying the aggregation of Aβ_42_ [30], it was depicted that the bulk of hydrophobic amino acid residues were confined inside the polypeptide chain in clustered polypeptides, bringing about the formation of three hydrophobic Aβ_42_ clusters. As these hydrophobic clusters extend through the fibril axis inside of packed subunits to be deposited away from solvents, they principally aid in keeping the protein fibril structure stable. By lessening free energy, hydrophobic contact is said to be the predominant force driving Aβ_42_ aggregation [30].

In our experiment, our pentapeptide TRRRR had hydrogen bond interactions with Val18 and Phe19 of the central hydrophobic core of the Aβ_42_ molecule. The pentapeptide ARRGR relatively interacted with 33 Gly, 33 Leu, and 35 Met through hydrogen bonds (Figure 1A,D). The pentapeptides were attached to similar hydrophobic regions as those already described by our laboratory [30,58]. When bound to the hydrophobic regions, they were able to obstruct and lessen the binding free energy between Aβ_42_ strands. The real inhibitory potencies of both TRRRR and ARRGR were probed by computational studies on Aβ_42_ and confirmed by in vitro studies. The ThT assay and transmission electron microscopy experiments elucidated that both TRRRR and ARRGR can successfully impede clusters of Aβ_42_. The fluorescent staining, MTT, and flow cytometric experiments further confirmed that the two pentapeptides reduced Aβ_42_ cytotoxicity (Figure 10 and Figure 11).

## 5. Conclusions

The present molecular dynamics and pharmacological experimental results indicate that TRRRR and ARRGR may bind to the hydrophobic clusters of the Aβ_42_ polymerization core to inhibit aggregation and ameliorate its neurotoxicity.

## Data Availability

All data related to the results presented in the paper are available upon request.

## Supplementary Materials

The following supporting information can be downloaded at: https://www.mdpi.com/article/10.3390/cimb46090606/s1, Supplementary Table S1. Docking free energy of pentapeptides to Aβ_42_.
