# Supplementary material for: Molecular Integrative Study on Inhibitory Effects of Pentapeptides on Polymerization and Cell Toxicity of Amyloid-β Peptide (1–42)
Source: Curr Issues Mol Biol. 2024 Sep 14;46(9):10160–79. doi: 10.3390/cimb46090606 (PMC11431437; doi:10.3390/cimb46090606)
Supplement: Supplementary file 1 [file cimb-46-00606-s001.zip › cimb-3158406-supplementary.pdf]

Supplementary Table S1. Docking free energy of pentapeptides to Aβ$_{42}$.

Ligand	△G*(kcal·mol^{-1})
TRRRR	-49.18
ARRGR	-41.39
RRRWR	-60.65
RRRDS	-46.28
TRRAR	-54.53

*△G was calculated using the generalized Born volume integral/weighted surface area method.
